# Mechanism of Neuronal versus Endothelial Cell Uptake of Alzheimer's Disease Amyloid β Protein

**DOI:** 10.1371/journal.pone.0004627

**Published:** 2009-02-27

**Authors:** Karunya K. Kandimalla, Olenych G. Scott, Smita Fulzele, Michael W. Davidson, Joseph F. Poduslo

**Affiliations:** 1 Department of Basic Pharmaceutical Sciences, College of Pharmacy and Pharmaceutical Sciences, Florida A&M University, Tallahassee, Florida, United States of America; 2 National High Magnetic Field Laboratory and Department of Biological Science, Florida State University, Tallahassee, Florida, United States of America; 3 Molecular Neurobiology Laboratory, Departments of Neurology, Neuroscience, and Biochemistry/Molecular Biology, Mayo Clinic College of Medicine, Rochester, Minnesota, United States of America; National Institutes of Health, United States of America

## Abstract

Alzheimer's disease (AD) is characterized by significant neurodegeneration in the cortex and hippocampus; intraneuronal tangles of hyperphosphorylated tau protein; and accumulation of β-amyloid (Aβ) proteins 40 and 42 in the brain parenchyma as well as in the cerebral vasculature. The current understanding that AD is initiated by the neuronal accumulation of Aβ proteins due to their inefficient clearance at the blood-brain-barrier (BBB), places the neurovascular unit at the epicenter of AD pathophysiology. The objective of this study is to investigate cellular mechanisms mediating the internalization of Aβ proteins in the principle constituents of the neurovascular unit, neurons and BBB endothelial cells. Laser confocal micrographs of wild type (WT) mouse brain slices treated with fluorescein labeled Aβ40 (F-Aβ40) demonstrated selective accumulation of the protein in a subpopulation of cortical and hippocampal neurons via nonsaturable, energy independent, and nonendocytotic pathways. This groundbreaking finding, which challenges the conventional belief that Aβ proteins are internalized by neurons via receptor mediated endocytosis, was verified in differentiated PC12 cells and rat primary hippocampal (RPH) neurons through laser confocal microscopy and flow cytometry studies. Microscopy studies have demonstrated that a significant proportion of F-Aβ40 or F-Aβ42 internalized by differentiated PC12 cells or RPH neurons is located outside of the endosomal or lysosomal compartments, which may accumulate without degradation. In contrast, BBME cells exhibit energy dependent uptake of F-Aβ40, and accumulate the protein in acidic cell organelle, indicative of endocytotic uptake. Such a phenomenal difference in the internalization of Aβ40 between neurons and BBB endothelial cells may provide essential clues to understanding how various cells can differentially regulate Aβ proteins and help explain the vulnerability of cortical and hippocampal neurons to Aβ toxicity.

## Introduction

Alzheimer's disease (AD), the most frequent form of senile dementia associated with progressive neurodegeneration, is characterized by extracellular amyloid plaques, intra-neuronal tangles, and cerebrovascular amyloid deposits. The extracellular plaques and cerebrovascular amyloid deposits contain amyloid β (Aβ) proteins, primarily Aβ40 and Aβ42, which are derived from the larger endogenously occurring amyloid precursor protein (APP). The extracellular amyloid plaques are predominantly formed in the hippocampus, cerebral cortex and other brain regions important for cognitive function; whereas, the cerebrovascular amyloid deposits are formed in the media and adventitia of small and mid-sized arteries and arterioles present in the cerebral cortex and leptomeninges, as well as cerebral capillaries, resulting in a condition known as cerebral amyloid angiopathy (CAA) [1]. Both AD and CAA are causatively linked. About 80% of AD patients were reported to manifest CAA [2].

Pathophysiological mechanisms resulting in amyloid accumulation in AD brain are poorly understood. While some researchers argue that the amyloid deposits are a mere downstream reflection of the neurodegeneration mediated by yet unidentified pathological events, others believe that Aβ is responsible for the neurodegeneration, and hence the plaques are central to the disease. Even though, the debate appears to be settling in favor of Aβ proteins as the root cause of AD pathology, one important question still lingers: whether extracellular Aβ deposition or intracellular Aβ accumulation initiates the AD process.

In a recent review, based on the biochemical, neuropathological and genetic information available till date, Wirths [3] indicated that Aβ accumulation in the neurons precedes the accumulation in the extracellular space and hypothesized that the intraneuronal Aβ accumulation is the first step of a fatal cascade of events leading to neurodegeneration in AD. The reports published by several other researchers strongly support this viewpoint. Mochizuki *et al.* [4] reported that cells, which were immunoreactive for Aβ42, colocalize with amyloid plaques in sporadic AD cases. Gouras et al. [5] demonstrated that the intraneuronal Aβ staining was most evident in the brain regions that show the first signs of plaque accumulation such as entorhinal cortex and hippocampus.

Upon accumulation, Aβ was reported to disrupt the normal functioning of neurons resulting in significant cellular dysfunction leading to apoptosis [6] and oxidative injury [7], even before the formation of senile plaques and neurofibrillary tangles. Significant neurodegeneration was reported in presenilin-1 (PS1) mutation bearing AD transgenic mice, which show extensive intraneuronal Aβ42 accumulation without any amyloid plaque formation in the brain [8]. Although not a good animal model for AD, the PS1 mice serves as a good example of the neuropathological consequences of intraneuronal Aβ. In addition to inducing neurodegeneration [9], intraneuronal Aβ aggregates may act as nidus for extracellular plaque formation, when Aβ-burdened neurons undergo lysis and the aggregates are released into the extracellular space [10].

Alongside the parenchymal amyloid plaques, AD patients exhibit vascular amyloid deposits to varying extent [11]. Deposition of amyloid in the cerebral vasculature results in thickening of the basal membrane, stenosis of the vessel lumen, and fragmentation of the internal elastic lamina, which may lead to stroke, brain hemorrhage, or dementia [12], [13]. Owing to the neurovascular etiology of AD [14], it is essential to interpret neurodegeneration caused by Aβ proteins in the perspective of vascular pathology. Uptake from the extracellular space, besides intraneuronal Aβ production, was hypothesized to be an important mechanism that contributes to Aβ accumulation in the neurons. Whereas, perturbed clearance across the blood-brain barrier (BBB) is believed to facilitate the formation of Aβ nidus that could eventually mature to cause CAA [15]. Therefore, knowledge of how neurons and BBB endothelial cells internalize extracellular Aβ proteins is an important prerequisite to deciphering the sequence of pathophysiological events causing these neurodegenerative diseases.

Published reports have proposed several pathways by which Aβ proteins can be internalized by neuronal and BBB endothelial cells. In neurons, the endocytosis of Aβ42 may be facilitated by the α7 nicotinic acetylcholine receptor [16] or NMDA receptor [11] expressed on the neuronal cell surface. In contrast, Aβ40 and 42 were reported to exhibit non-saturable uptake in differentiated PC12 cells [17] and human neuroblastoma cells [18]. Burdick et al. [17] reported that the accumulation of ^125^I labeled Aβ40 and Aβ42 proteins in PC12 cells at 4°C was resistant to trypsin digestion, indicating that their uptake is energy-independent. In the blood-brain-barrier (BBB) endothelial cells, the internalization of Aβ40 was claimed to be mediated by receptor for advanced glycosylated end products (RAGE) at the luminal surface [19] and LRP1 at the abluminal surface [20].

These reports imply that neurons and BBB endothelial cells internalize Aβ40 and 42 via different portals, and the factors governing internalization in these two cell types could be substantially different. Therefore, mechanistic details of Aβ protein internalization mediated by a variety of receptors expressed in these cell types must be adequately resolved to estimate their impact on Aβ accumulation and/or clearance. In addition, the nature of energy independent and non-endocytotic processes proposed to play a role in the neuronal uptake of Aβ40 and 42 need to be thoroughly explored. Biophysical studies conducted on phospholipid bilayers and in vesicles made of total brain lipids demonstrated the ability of Aβ40 and 42 to intercalate into the phospholipid bilayer of the plasma membrane [21]. Whether this unique behavior enables Aβ proteins to passively diffuse across the plasma membrane and reach the cytoplasm, needs to be carefully examined. The current study is aimed at investigating some of these aspects in neuronal and BBB endothelial cell models.

## Results

### Uptake of Aβ40 in wild type (WT) mouse brain slices

**Figure 1:**
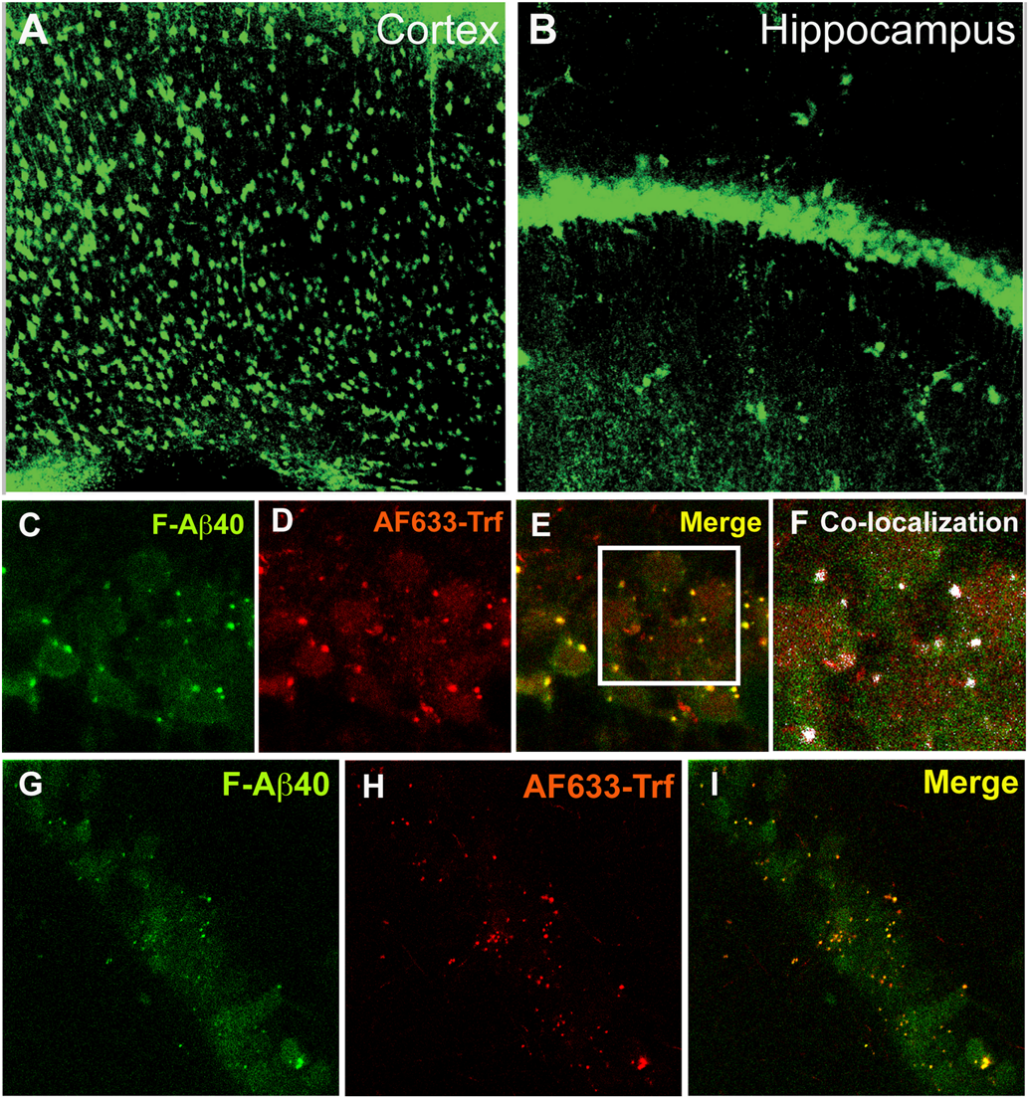
Cellular uptake of a fluorescein labeled Aβ40 (F-Aβ40) and Alexa Fluor® 633 labeled transferrin (AF633-Trf) in wild type mouse brain slices after 30 min incubation. A–B: Uptake of F-Aβ40 by a subpopulation of (A) cortical neurons (20×) and; (B) hippocampal neurons (20×). C–I: Effect of temperature on the uptake of F-Aβ40 and AF633-Trf by the pyramidal neurons (63× and 3× optical zoom). C–F: Uptake of F-Aβ40 and AF633-Trf at 37°C (C) F-Aβ40 uptake; (D) AF633-Trf uptake; (E) Superimposition of images C and D; (F) Limited co-localization of F-Aβ40 and AF633-Trf, indicated by white masked areas, was found only around the edges of pyramidal neurons. G–I: Uptake of F-Aβ40 and AF633-Trf at 4°C (G) F-Aβ40 uptake; (H) Inhibition of AF633-Trf internalization; (I) Superimposition of images G and H.

While, extensively used in neurophysiology research [22], murine brain slices are also employed to study diffusion of drugs in the brain tissue [23], [24] because they provide a physiological environment with an intact cyto-architecture consisting of natural extracellular matrix, neuronal connectivity, and intercellular interactions. The confocal micrographs of WT mouse brain slices, imaged after a 30 min incubation in oxygenated Krebs-Ringer bicarbonate buffer (KRB) containing fluorescein labeled Aβ40 (F-Aβ40) and Alex Fluor633 labeled transferrin (AF633-Trf) followed by a quick wash with acidified KRB and then phosphate buffered saline (PBS), demonstrated F-Aβ40 accumulation in a subpopulation of cortical (**Figure 1A**) and hippocampal (**Figure 1B**) cells. Based on the size and morphology, these cells were identified as neurons. In addition to F-Aβ40, AF633-Trf (endocytotic marker whose cellular internalization is energy dependent) was also internalized by hippocampal neurons. A careful visual examination of the images in Figure 1(C–E) reveals punctate bright localization of Aβ40 and AF633-trf around the edges of the neuronal cell body, but the center of the cell harbors a fainter signal of the fluorophores. The co-localization of the fluorophores, indicated by the white masked areas, was mostly limited to the outer edges of the cell; whereas very limited co-localization was noticed towards the center of the cell at 37°C (**Figure 1F**).

To elucidate the role of energy in the internalization of these fluorophores by hippocampal neurons, the above experiments were repeated at 4°C, which inhibits most of the physiological processes including the cellular transport and metabolism of proteins. At 4°C, bright F-Aβ40 and AF633-trf signal around the edges of the neuronal cell body was not affected. But the intensity of AF633-trf signal distributed towards the center of the cell body decreased significantly. Surprisingly, F-Aβ40 signal in the same cellular region was not significantly affected at 4°C (**Figure 1G–I**). Moreover, F-Aβ40 maintained similar cellular localization patterns at 37°C and 4°C (**Figure 1G**).

**Figure 2:**
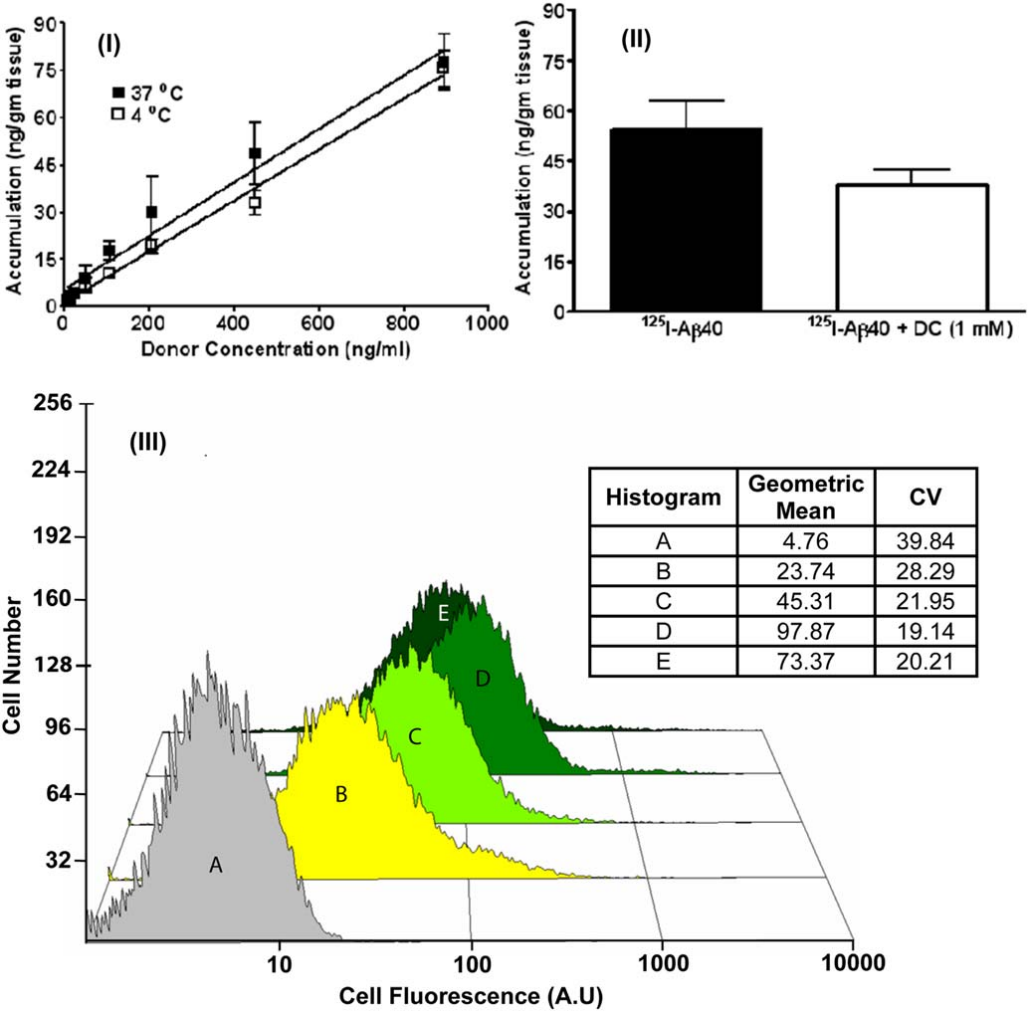
(I) Effect of donor concentration and temperature on the uptake of ^125^I-Aβ40 in wild type (WT) mouse brain slices. (II) Effect of endocytotic inhibitor dansyl cadaverine on the uptake of ^125^I-Aβ40 (450 ng/ml) in WT mouse brain slices. (III) Histograms of fluorescence intensity in differentiated PC12 cells exposed to various concentrations of F-Aβ40. (A) Untreated cells; (B) Cells incubated with 0.65 µM F-Aβ40; (C) Cells incubated with 1.3 µM F-Aβ40; (D) Cells incubated with 3.2 µM F-Aβ40; (E) Cells incubated with 3.2 µM F-Aβ40+32 µM unlabeled Aβ40.

We have also utilized the brain slices model to quantify cellular uptake of Aβ40 at donor concentrations relevant to AD pathophysiology. Due to the assay limitations with F-Aβ40 at lower concentrations, ^125^I labeled Aβ40 (^125^I-Aβ40) was employed in these studies. The uptake of ^125^I-Aβ40 in WT mouse brain slices at 4 or 37°C was determined following 15 min incubation in oxygenated KRB containing 5–900 ng/ml (0.02–3.5 µCi/ml) concentrations of ^125^I-Aβ40. The 15 min incubation time was selected based on our earlier observations that ^125^I-Aβ40 uptake in the brain slices was linear up to 15 min, but reached a plateau between 15 and 60 min [25]. Accumulation of ^125^I-Aβ40 in the brain slices was linearly dependent upon the donor concentration (**Figure 2I**). In addition, no significant differences were observed between the uptake of ^125^I-Aβ40 at 37 and 4°C (**Figure 2I**). Moreover, ^125^I-Aβ40 uptake in the brain slices treated with 1 mM dansyl cadaverine (DC), an endocytotic inhibitor, was not significantly affected (**Figure 2II**).

These intriguing observations suggest non-endocytotic uptake of Aβ40 which was further examined in differentiated rat pheochromocytoma cells (PC12), a neuronal cell culture model widely used for studying various neurophysiological processes, including intra-neuronal protein trafficking [26], [27]. Attempts made a decade ago to determine the uptake of ^125^I-Aβ40 in differentiated PC12 cells lead to an understanding that the protein is internalized even at 4°C [17]. In addition, our anlaysis of the published data [17] indicated that the internalization of ^125^I-Aβ40 by differentiated PC12 cells is linearly dependent upon the donor concentration, which coincided with the current observations made in murine brain slices model. Further experimentation with ^125^I-Aβ40 is unlikely to provide additional information towards mechanistic understanding of the uptake phenomenon. Therefore, in the current study we employed F-Aβ40 to take advantage of the current advances in optical microscopy and the availability of probes to track the protein accumulation in various cellular organelles. Moreover, a quantitative determination of F-Aβ40 internalization in differentiated PC12 cells can be made with the help of flow cytometry techniques.

The effect of donor concentration on the uptake of F-Aβ40 by differentiated PC12 cells was determined by flow cytometry. The resultant histograms of cellular fluorescence indicated that the accumulation of F-Aβ40 was linear with the donor concentrations ranging between 3 and 15 µg/ml (**Figure 2III A–D**). To evaluate the saturability of uptake at higher donor concentrations, the cells were co-incubated with 150 µg/ml of unlabeled Aβ40 and 15 µg/ml of F-Aβ40. The fluorescence intensity in these cells decreased compared to the cells treated with solutions containing 15 µg/ml of F-Aβ40 alone (**Figure 2III E**).

### Localization of F-Aβ40 in the acidic compartments of differentiated PC12 cells

**Figure 3:**
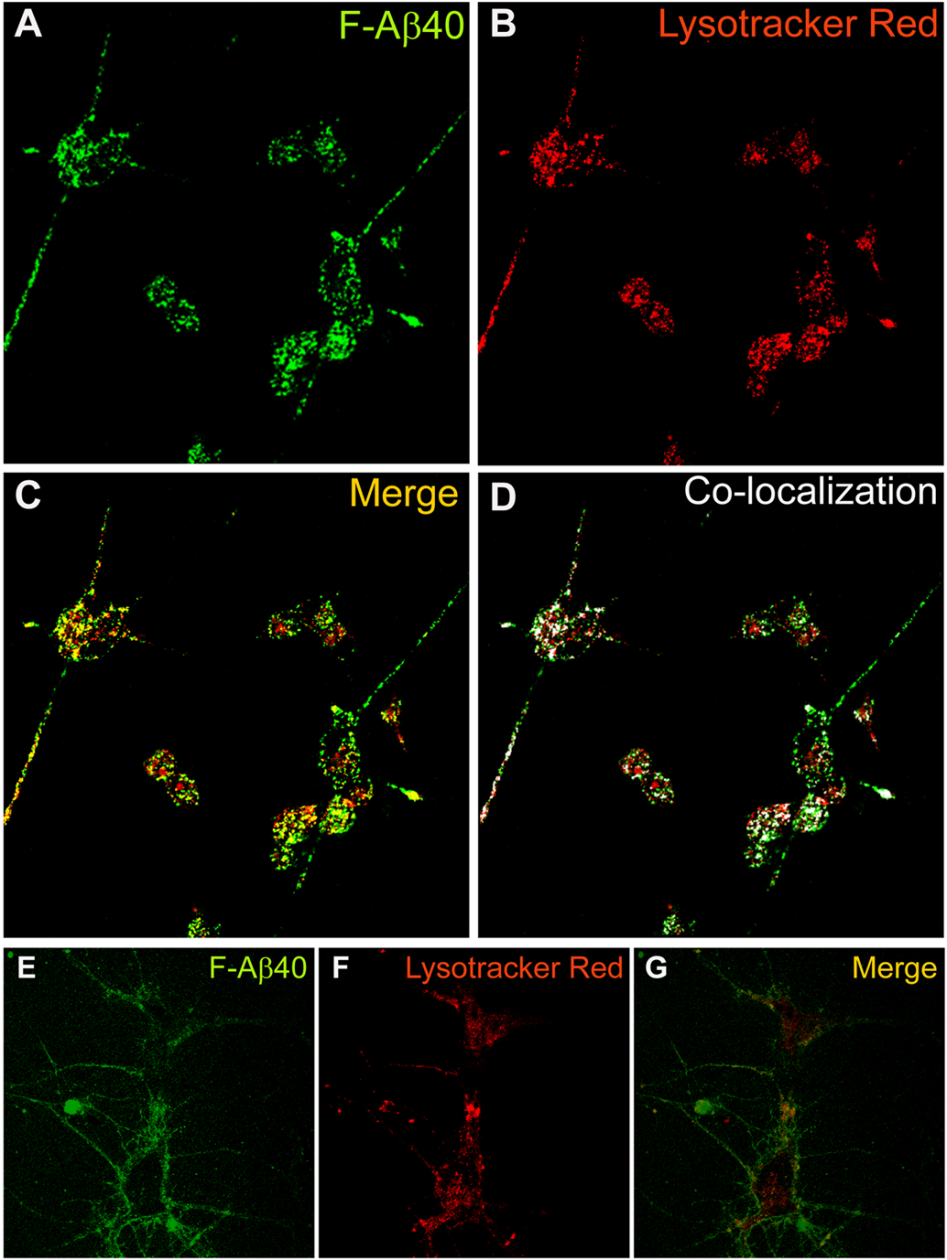
A–D: Extent of fluorescein labeled Aβ40 (F-Aβ40) uptake in the acidic compartments of differentiated PC12 cells labeled by Lysotracker Red® (40×); (A) F-Aβ40 uptake (B) Uptake of Lysotracker Red®; (C) Superimposition of images A and B; (D) Partial co-localization of Lysotracker Red® and F-Aβ40 indicated by white masked areas. E–G: Extent of F-Aβ40 uptake in the acidic compartments of rat primary hippocampal (RPH) neurons labeled by Lysotracker Red® (40×); (E) F-Aβ40 uptake (F) Uptake of Lysotracker Red® Red; (G) Superimposition of images E and F.

Several researchers in the past have claimed that Aβ40 is internalized by neurons and blood brain barrier (BBB) endothelial cells via receptor mediated endocytosis [16], [19], [20]. The sub-cellular itinerary of an endocytosed protein usually involves accumulation in acidic cell compartments, including early endosomes, late endsomes, and lysosomes. Differentiated PC12 cells incubated with F- Aβ40 (**Figure 3A**) and LysoTracker red® (**Figure 3B**), a fluorophore that selectively labels acidic compartments (primarily late endosomes and lysosomes) of living cells, demonstrated only a partial co-localization of both fluorophores (**Figure 3C–D**). A similar phenomenon was also observed in rat primary hippocampal (RPH) neurons (**Figure 3E–G**), which accumulated Lysotracker Red® predominantly in the cell body (**Figure 3F**), but F-Aβ40 in the neurites (**Figure 3E**). These results showed that only a portion of internalized F-Aβ40 cycles through the endosomes and lysosomes of PC12 cells and RPH neurons, but a noticeable portion accumulates outside of these acidic compartments.

### Role of endocytosis in the uptake of F-Aβ40 by differentiated PC12 cells

**Figure 4:**
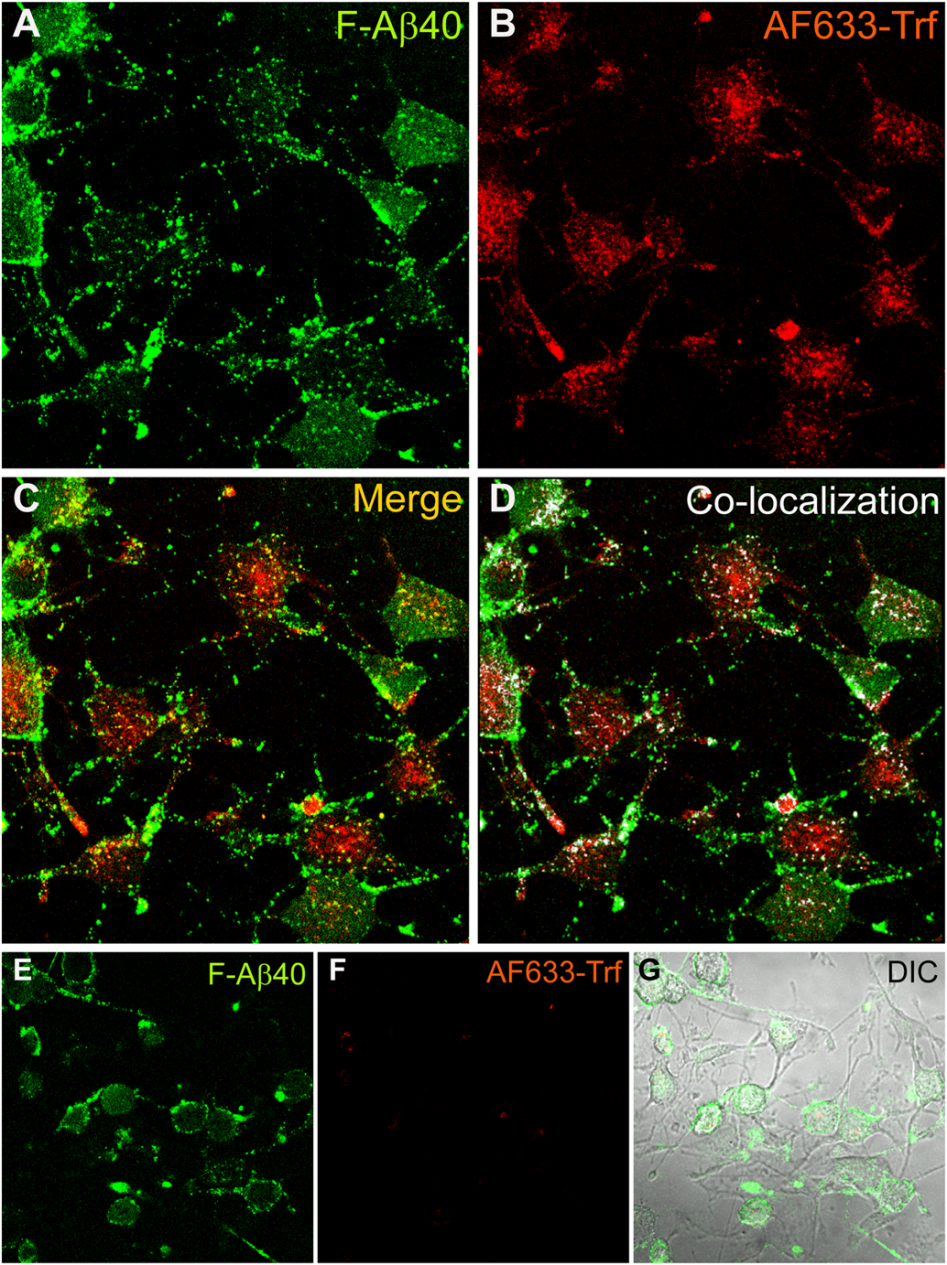
A–D: Uptake of fluorescein labeled Aβ40 (F-Aβ40) and Alexa Fluor® 633 labeled transferrin (AF633-Trf), clathrin-mediated endocytosis marker, by differentiated PC12 cells following 30 min incubation. (A) F-Aβ40 uptake; (B) Uptake of AF633-Trf; (C) Superimposition of images A and B; (D) Sparse co-localization of F-Aβ40 and AF633-Trf as indicated by the white masked areas. E–G: Uptake of F-Aβ40 and AF633-Trf in differentiated PC12 cells subjected conditions that inhibit clathrin mediated endocytosis (hypotonic shock for 5 min followed by incubation with potassium free salt solution for 30 min). (E) Uptake of F-Aβ40; (F) Substantial reduction in AF633-Trf; (G). Superimposition of images E and F on the differential interference contrast (DIC) image to show the condition of the cells.

**Figure 5:**
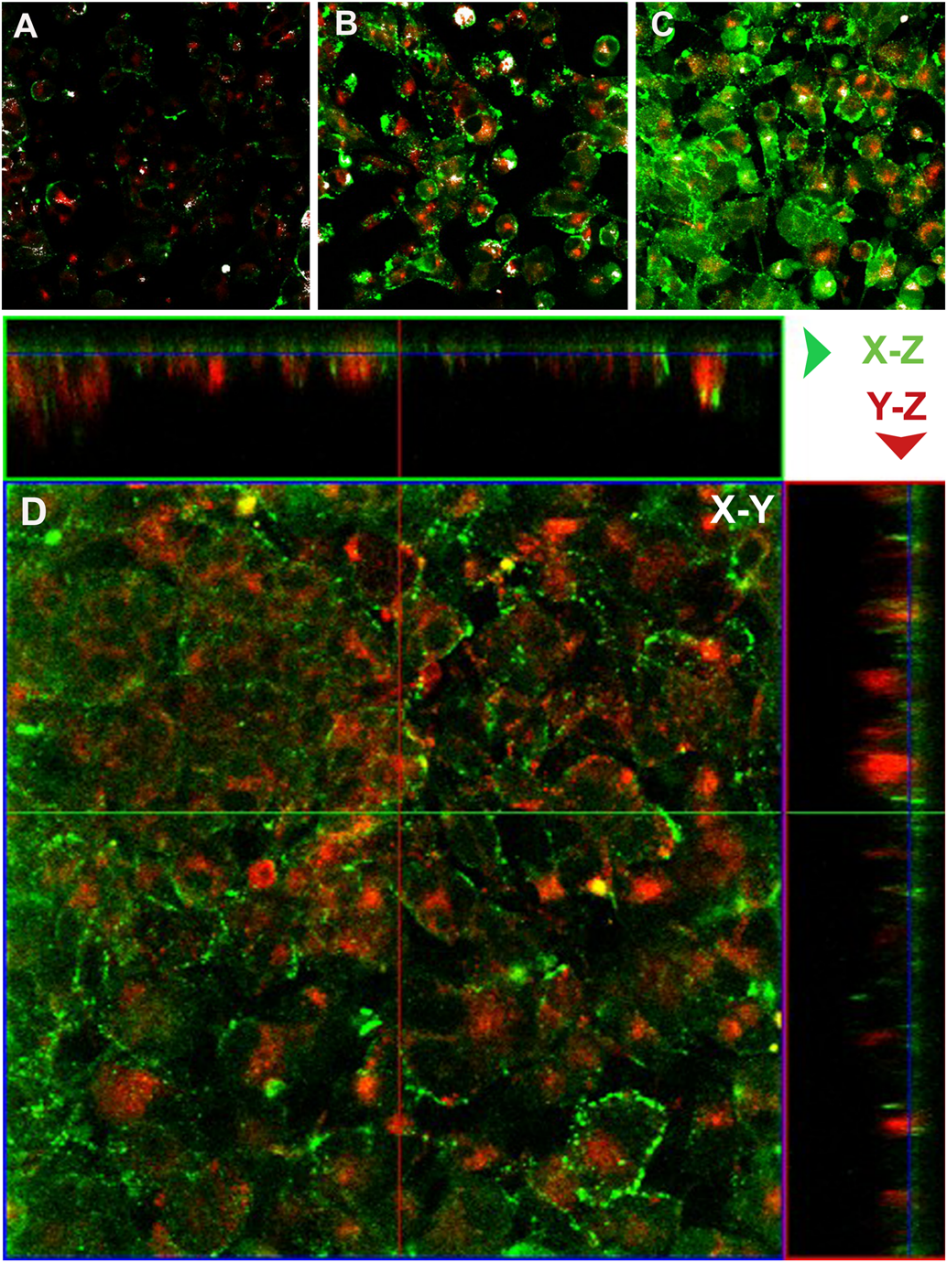
A–C: Co-localization of fluorescein labeled Aβ40 (F-Aβ40) and Alexa Fluor® 633 labeled transferrin (AF633-Trf) in PC12 cells following: (A) 15 min; (B) 45 min; and (C) 60 min incubation. White masked areas indicate the extent of co-localization of F-Aβ40 and AF633-Trf. D: Cellular internalization of F-Aβ40 and AF633-Trf established by the XY, XZ, and YZ projections of differentiated PC12 cells treated with the fluorophores for 60 min. Optical sections (planes 1–45) were obtained from the coverslip bottom to the cell surface with a 0.6 µm Z-step interval.

Following the incubation of differentiated PC12 cells with F-Aβ40 and AF633-Trf, a punctate localization of both proteins was observed in the perinuclear region (**Figure 4A&B**). Composite image generated by superimposing F-Aβ40 and AF633-Trf images (**Figure 4C**) as well as the co-localization map of F-Aβ40 and AF633-Trf overlapping pixel regions, represented by white masked areas (**Figure 4D**), demonstrated little co-localization of the fluorophores. Hypotonic shock followed by incubation in potassium depleted salt solution was shown to inhibit clathrin-mediated endocytosis [28]. Under these conditions, the uptake of F-Aβ40 was not affected (**Figure 4E**) but the uptake of AF633 Trf, which is internalized via clathrin mediated endocytosis, was significantly diminished (**Figure 4F**). Differential Interference Contrast (DIC) image indicates that the cell morphology was not significantly altered under these experimental conditions (**Figure 4G**). Due to dynamic nature of AF633-Trf internalization, the co-localization of F-Aβ40 with the endocytotic marker was also determined at various time points of 15, 45, and 60 min following the incubation. The resultant co-localization maps, presented as figures 5A (15 min), 5B (45 min), and 5C (60 min), did not reflect major shifts in the co-localization patterns with time. However, the location of F-Aβ40 accumulation in the cells, but not of AF633-Trf, changed significantly with time. Following 15 min incubation, AF633-Trf appeared to accumulate in the juxta-nuclear region of the PC12 cells, whereas F-Aβ40 was mainly confined to the cell membrane. At the end of 45 min, F-Aβ40 appeared to move into the cytosol; and by the end of 60 min, the F-Aβ40 intensity in the cells increased substantially. To differentiate the pools of F-Aβ40 internalized by the cells from that bound to the cell membrane, z-series confocal images of PC12 cells incubated with F-Aβ40 and AF633-Trf for 60 min were obtained and presented as XY, XZ, and YZ projections (Figure 5D).

**Figure 6:**
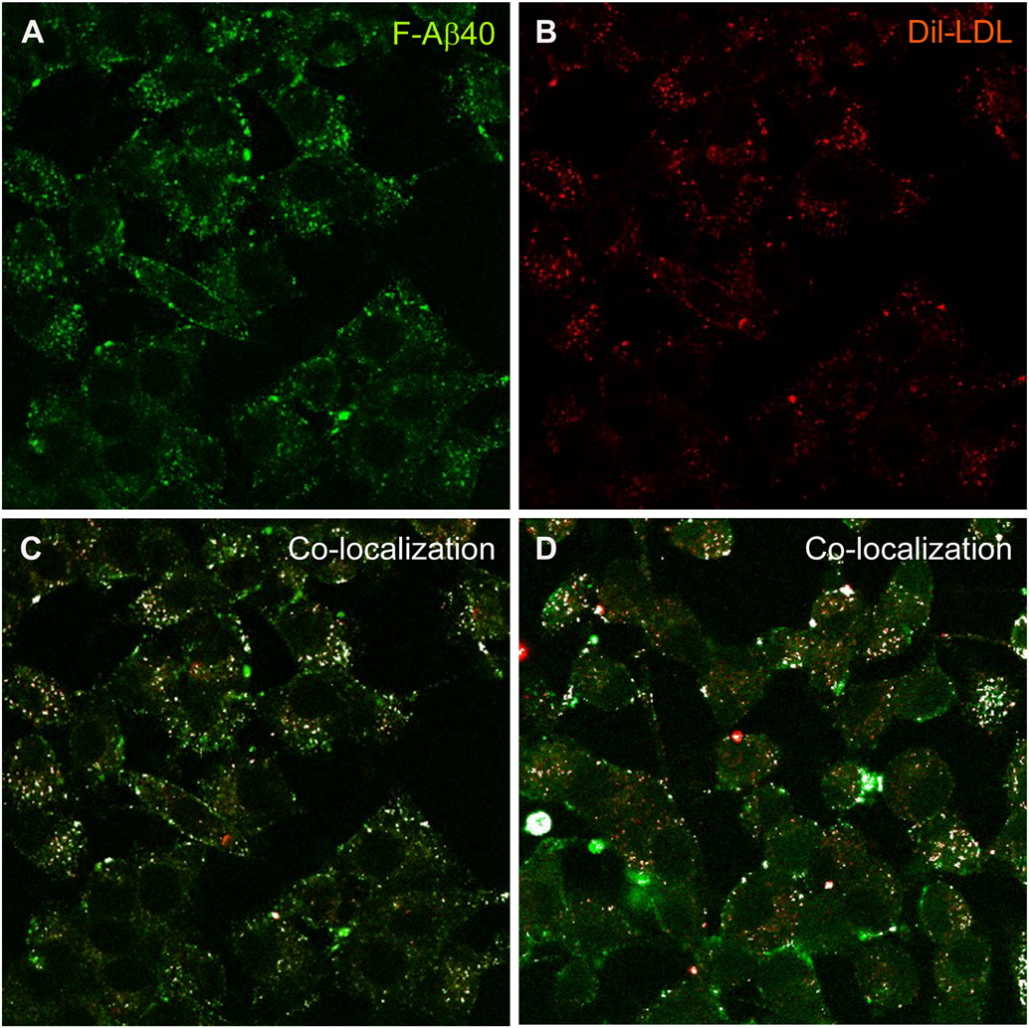
A–C: Accumulation of fluorescein labeled Aβ40 (F-Aβ40) and Dil labeled low density lipoprotein (Dil-LDL) in the late endosomes of differentiated PC12 cells following a 15 min treatment. (A) F-Aβ40 uptake; (B) Uptake of Dil-LDL; (C) Superimposition of images A and B indicate only a partial co-localization, shown by the white masked areas. D: Co-localization of F-Aβ40 and Dil-LDL in differentiated PC12 cells following a 30 min treatment.

The endocytotic marker used in these studies, AF633-Trf, localizes primarily in early endosomes. Therefore, similar uptake studies were conducted using Dil labeled low density lipoprotein complex (Dil-LDL) to capture the accumulation of F-Aβ40 in the late endosomes (Figure 6A–D). These studies also demonstrated a partial co-localization of F-Aβ40 with Dil-LDL at both 15 (Figure 6C) and 30 min (Figure 6D) following incubation.

**Figure 7:**
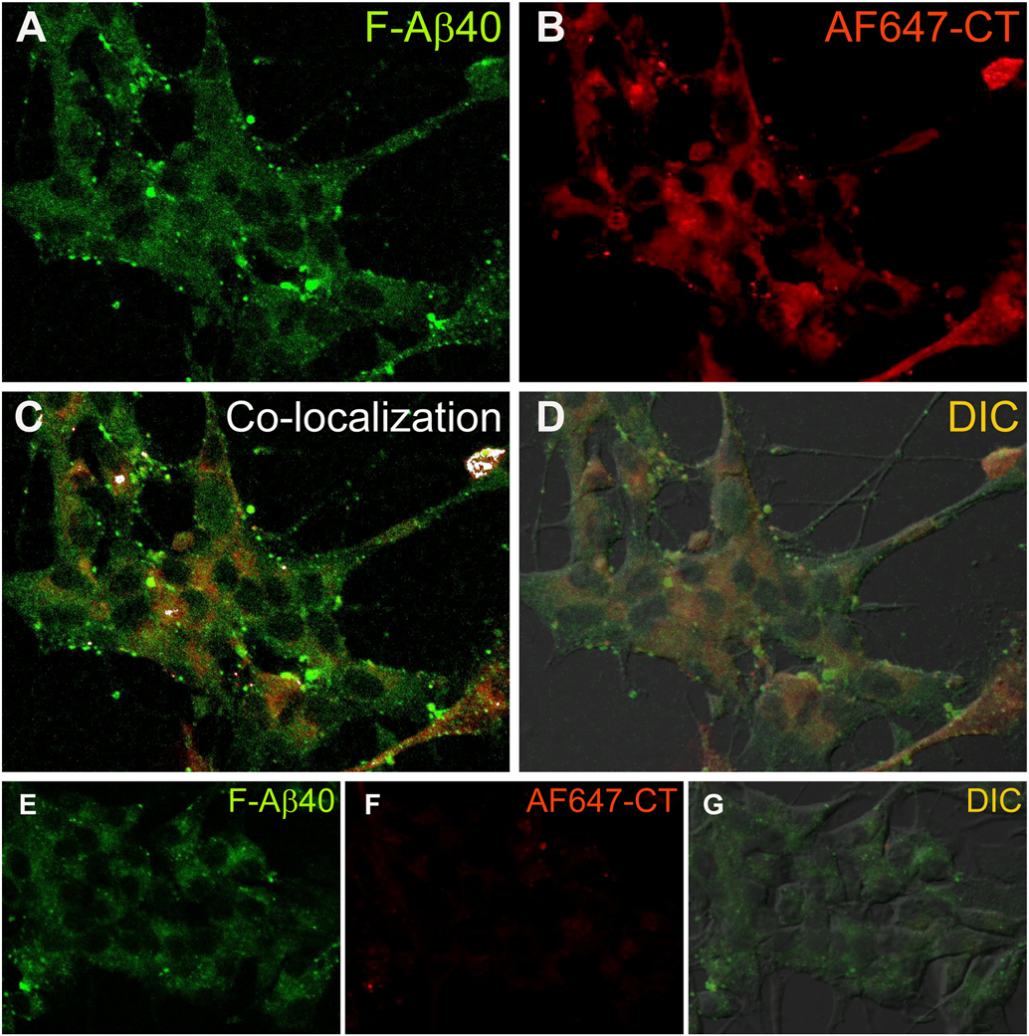
A–D: Uptake of fluorescein labeled Aβ40 (F-Aβ40) and Alexa Fluor® 647 labeled cholera toxin (AF647-CT), caveolae-mediated endocytosis marker, by differentiated PC12 cells following 30 min incubation. (A) F-Aβ40 uptake; (B) Uptake of AF647-CT; (C) Sparse co-localization of F-Aβ40 and AF647-CT as indicated by the white masked areas; (D) Superimposition of images A and B on the differential interference contrast (DIC) image to show the location of the fluorophores in the cells. E–G: Uptake of F-Aβ40 and AF647-CT in differentiated PC12 cells treated with filipin, which is known to inhibit caveolae mediated endocytosis. (E) Uptake of F-Aβ40; (F) Substantial reduction in AF647-CT uptake in the cells treated with filipin; (G). Superimposition of images E and F on the differential interference contrast (DIC) image to show the health of filipin treated cells.

PC12 cells incubated with 15 µg/ml F-Aβ40 and 5 µg/ml Alexa Fluor 647 cholera toxin (AF647-CT), a marker for caveolae mediated endocytosis, exhibited punctuate localization of F-Aβ40 (**Figure 7A**) but AF647-CT fluorescence diffused throughout the perinuclear region (**Figure 7B**). Both fluorophores co-localized very slightly (**Figure 7C–D**). The uptake of AF633-CT in PC12 cells treated with filipin, an inhibitor of caveolae mediated endocytosis [29], was severely impaired (**Figure 7F**), whereas the uptake of F-Aβ40 was not significantly affected (**Figure 7E**). The DIC image (**Figure 7G**) showed well preserved cell morphology.

These studies clearly demonstrated lack of co-localization of F-Aβ40 with clathrin or caveolae mediated endocytosis markers. Moreover, the conditions that were known to reduce these endocytotic processes did not affect the internalization of F-Aβ40 significantly.

### Role of temperature and cellular ATP in the uptake of F-Aβ40 by PC12 cells

Differences in the internalization of F-Aβ40 and AF633-Trf, controlled for temperature and energy dependent transport, was also assessed using laser confocal microscopy and flow cytometry.

#### Internalization of F-Aβ40 and AF633-Trf at 4°C

**Figure 8:**
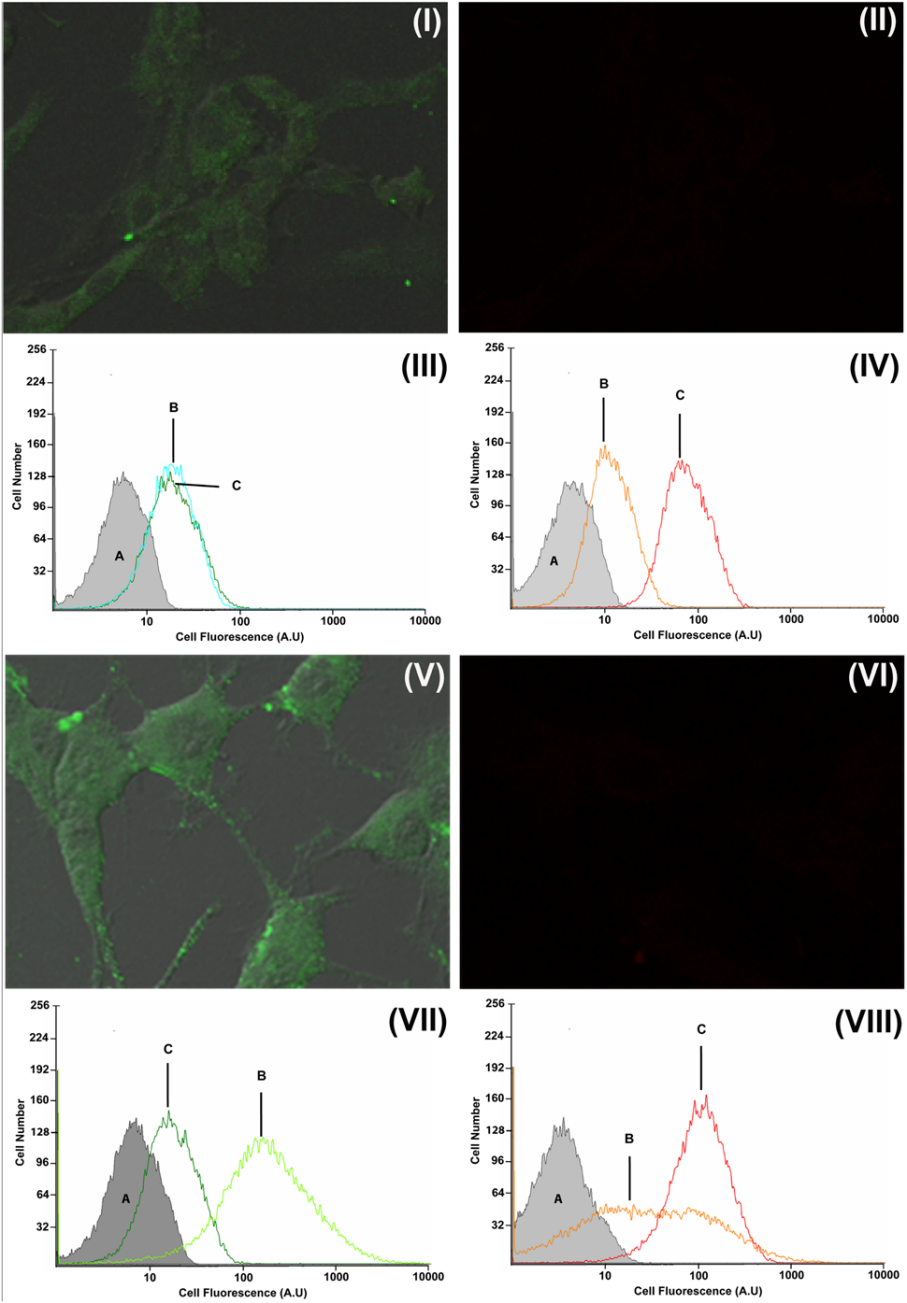
I–IV: Uptake of fluorescein labeled Aβ40 (F-Aβ40) and Alexa Fluor® 633 labeled transferrin (AF633-Trf) in differentiated PC12 cells at 4°C. (I) Uptake of F-Aβ40 (20×); (II) Uptake of AF633-Trf (20×). III–IV: Histograms of fluorescence intensity in differentiated PC12 cells treated with: (III) F-Aβ40: (A) Untreated control, (B) At 4°C, (C) At 37°C; and (IV) AF633-Trf: (A) Untreated control, (B) At 4°C, (C) At 37°C. V–VIII: Uptake of F-Aβ40 and AF633-Trf in differentiated PC12 cells treated with 10 mM Sodium Azide and 50 mM 2-deoxy glucose, agents that are known to deplete cellular ATP. (V) Uptake of F-Aβ40 (40×); (VI) Uptake of AF633-Trf (40×). VII–VIII: Histograms of F-Aβ40 fluorescence intensity in differentiated PC12 cells (VII) F-Aβ40: (A) Untreated control, (B) ATP depleted cells, (C) Normal cells; and (VIII) AF633-Trf: (A) Untreated control; (B) ATP depleted cells; (C) Normal cells.

Laser confocal micrographs of the PC12 cells incubated with F-Aβ40 and AF633-Trf at 4°C showed vesicular localization of F-Aβ40 (**Figure 8I**), but the fluorescence intensity reduced compared to that at 37°C. As expected, these cells did not show detectable levels of AF633-Trf (**Figure 8II**). The histograms of cellular fluorescence obtained from flow cytometry analysis demonstrated that the uptake of F-Aβ40 at 4°C (**Figure 8III B**) was not significantly different from the uptake at 37°C (**Figure 8III C**); but the uptake of AF633-Trf at 4°C (**Figure 8IV B**) was significantly lower than the uptake at 37°C (**Figure 8IV C**).

#### Internalization of F-Aβ40 and AF633-Trf under ATP depleted conditions

The PC12 cells depleted of ATP showed significantly greater uptake of F-Aβ40 than the normal cells (**Figure 8V**) at 37°C. However, no detectable AF633-Trf signal was observed in these cells (**Figure 8VI**). Based on the DIC and fluorescence image composite, the cells depleted of ATP appeared normal at the end of the experiment (**Figure 8V**). The results from flow cytometry studies conducted on ATP depleted cells were in agreement with the observations made in the microscopy studies. In the PC12 cells depleted of cellular ATP, the uptake of F-Aβ40 at 37°C (**Figure 8VII B**) was significantly higher than that of the normal cells (**Figure 8VII C**). The uptake of AF633-Trf, on the other hand, was significantly impaired in ATP depleted cells (**Figure 8VIII B**).

These results clearly demonstrated that the PC12 cells simultaneously treated with F-Aβ40 and AF633-Trf at 37°C accumulated both the fluorophores without noticeable co-localization. However, at 4°C or under ATP depleted conditions, which inhibit active transport processes including receptor mediated endocytosis, F-Aβ40 internalization by PC12 cells was not inhibited whereas the receptor mediated endocytosis of AF633-Trf was substantially impaired.

### Role of endocytosis in the internalization of F-Aβ42 by differentiated PC12 cells

**Figure 9:**
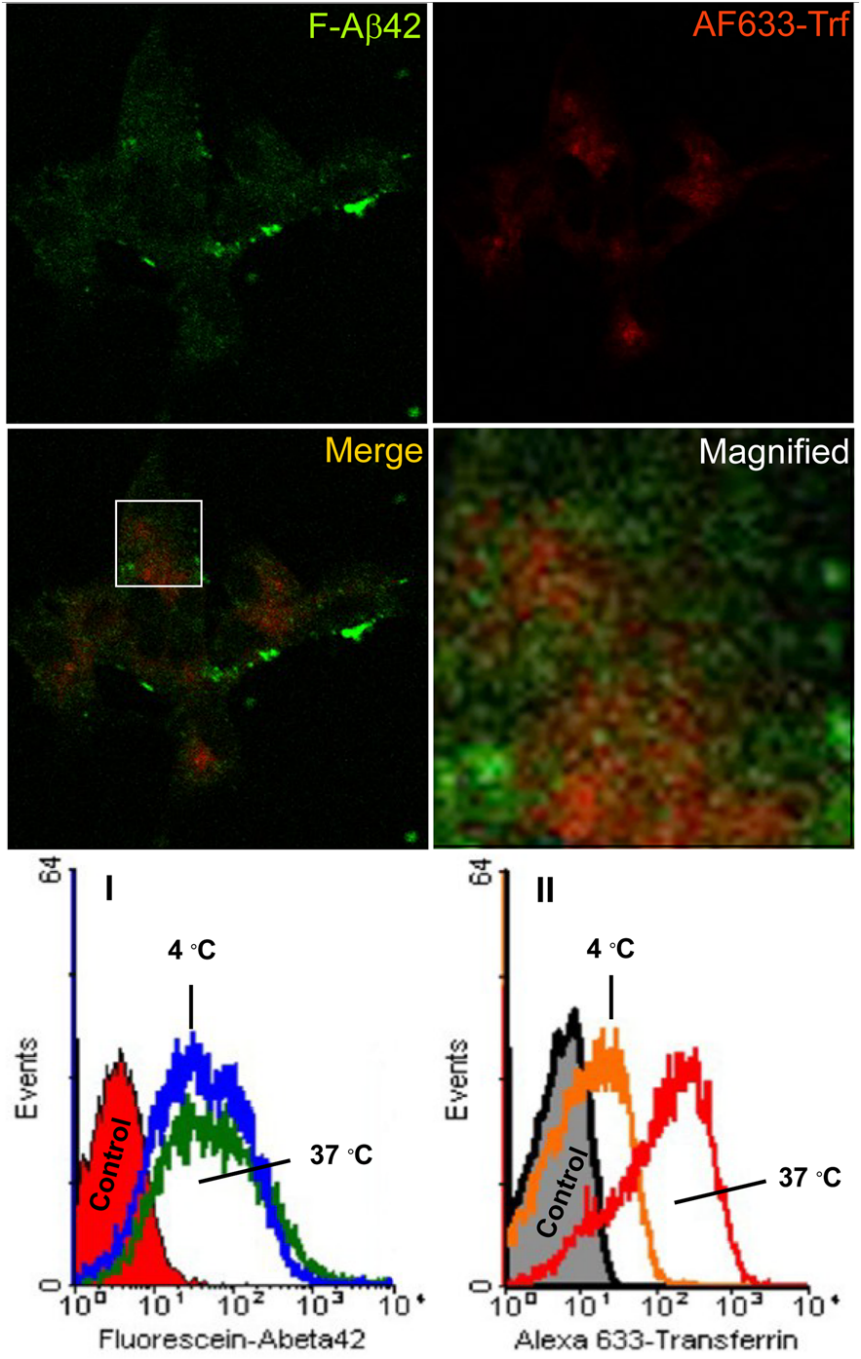
A–D: Uptake of fluorescein labeled Aβ42 (F-Aβ42) and Alexa Fluor® 633 labeled transferrin (AF633-Trf), clathrin-mediated endocytosis marker, by differentiated PC12 cells following 30 min incubation. (A) F-Aβ42 uptake; (B) Uptake of AF633-Trf; (C) Superimposition of images A and B show limited co-localization of F-Aβ42 and AF633-Trf. (D) A magnified portion of image C (enclosed in the white rectangle) to indicate the lack of co-localization of both fluorophores. I–II: Histograms of fluorescence intensity in differentiated PC12 cells treated with (I) F-Aβ42; and (II) AF633-Trf, at 37°C and 4°C.

Following the incubation with F-Aβ42 and AF633-Trf, the PC12 cells internalized both proteins in the perinuclear region (**Figure 9A&B**). A Composite of green and red channel images demonstrated little co-localization of the fluorophores (**Figure 9C**), which is also confirmed by the magnified inset (**Figure 9D**) of the composite image. The histograms of cellular fluorescence obtained from flow cytometry analysis demonstrated that the uptake of F-Aβ42 at 4°C was not significantly different from that at 37°C (**Figure 9I**); but the uptake of AF633-Trf at 4°C was significantly lower than the uptake at 37°C (**Figure 9II**).

### Uptake of F-Aβ40 in rat primary hippocampal (RPH) neurons

**Figure 10:**
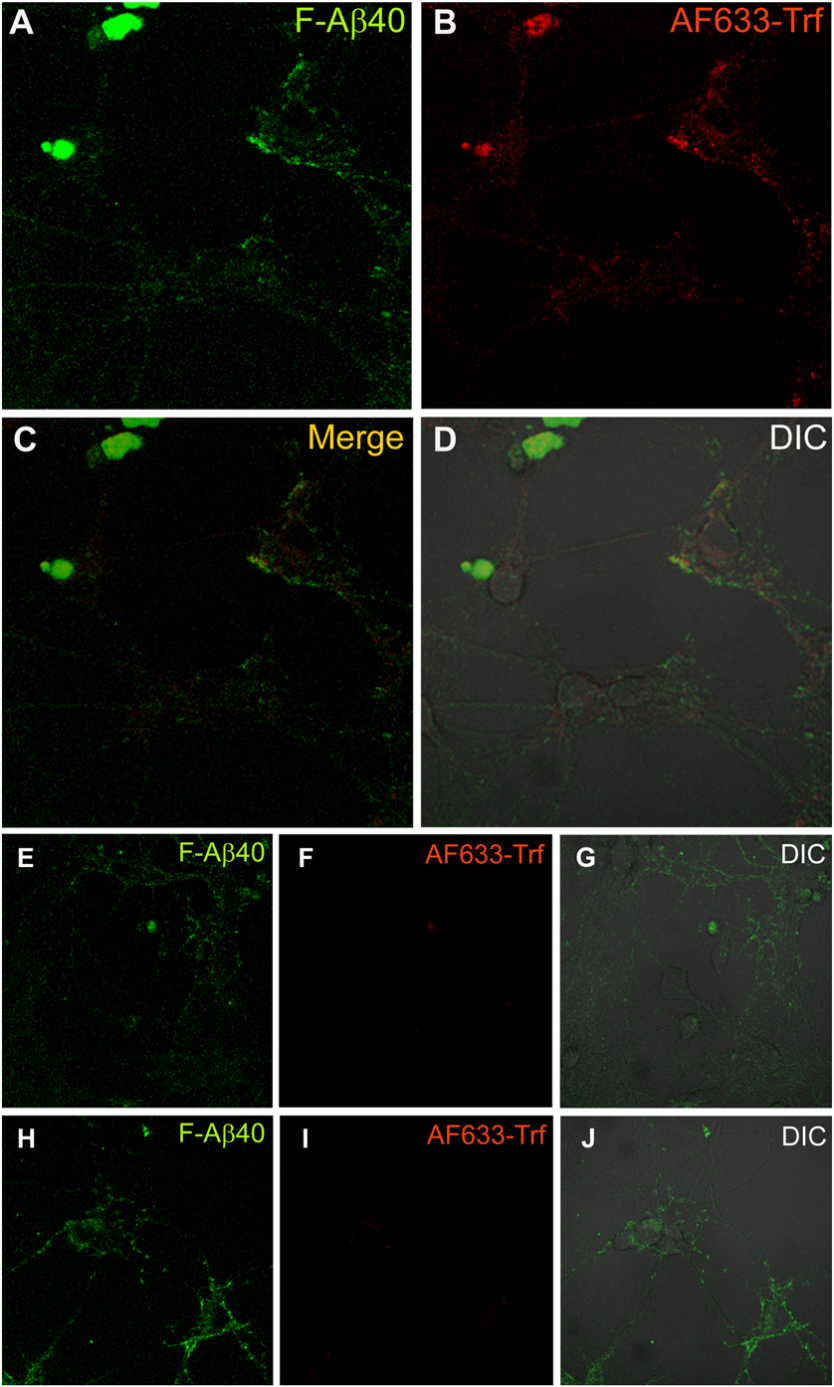
A–D: Uptake of fluorescein labeled Aβ40 (F-Aβ40) and Alexa Fluor® 633 labeled transferrin (AF633-Trf), clathrin-mediated endocytosis marker, in rat primary hippocampal (RPH) neurons following 30 min incubation at 37°C. (A) F-Aβ40 uptake; (B) Uptake of AF633-Trf; (C) Superimposition of images A and B; (D) Overlay of fluorescence images on the DIC image of RPH neurons. E–G: Uptake of F-Aβ40 and AF633-Trf in RPH neurons at 4°C. (E) Uptake of F-Aβ40; (F) No significant neuronal uptake of AF633-Trf at 4°C; (G) Superimposition of images D and E on the DIC image of RPH neurons; H–J: Uptake of F-Aβ40 and AF633-Trf in RPH neurons treated with 10 mM Sodium Azide and 50 mM 2-deoxy glucose, agents that are known to deplete cellular ATP. (H) Uptake of F-Aβ40; (I) No significant cellular uptake of AF633-Trf was observed; (J) Superimposition of images H and I on the DIC image of neurons.

Observations made in PC12 cells and adult hippocampal neurons (WT mouse brain slices) were verified in RPH neurons. At 37°C, RPH neurons internalized F-Aβ40 as well as AF633-Trf (**Figure 10A–B**) without significant co-localization (**Figure 10C–D**). The uptake of AF633-Trf reduced significantly in the RPH neurons incubated at 4°C (**Figure 10F**), whereas, the uptake of F-Aβ40 was not affected (**Figure 10E**). In RPH neurons depleted of cellular ATP, the uptake of F-Aβ40 increased considerably (**Figure 10H**), whereas no detectable uptake of AF633-Trf (**Figure 10I**) was observed.

### Uptake of F-Aβ40 in bovine brain microvascular endothelial (BBME) cells

**Figure 11:**
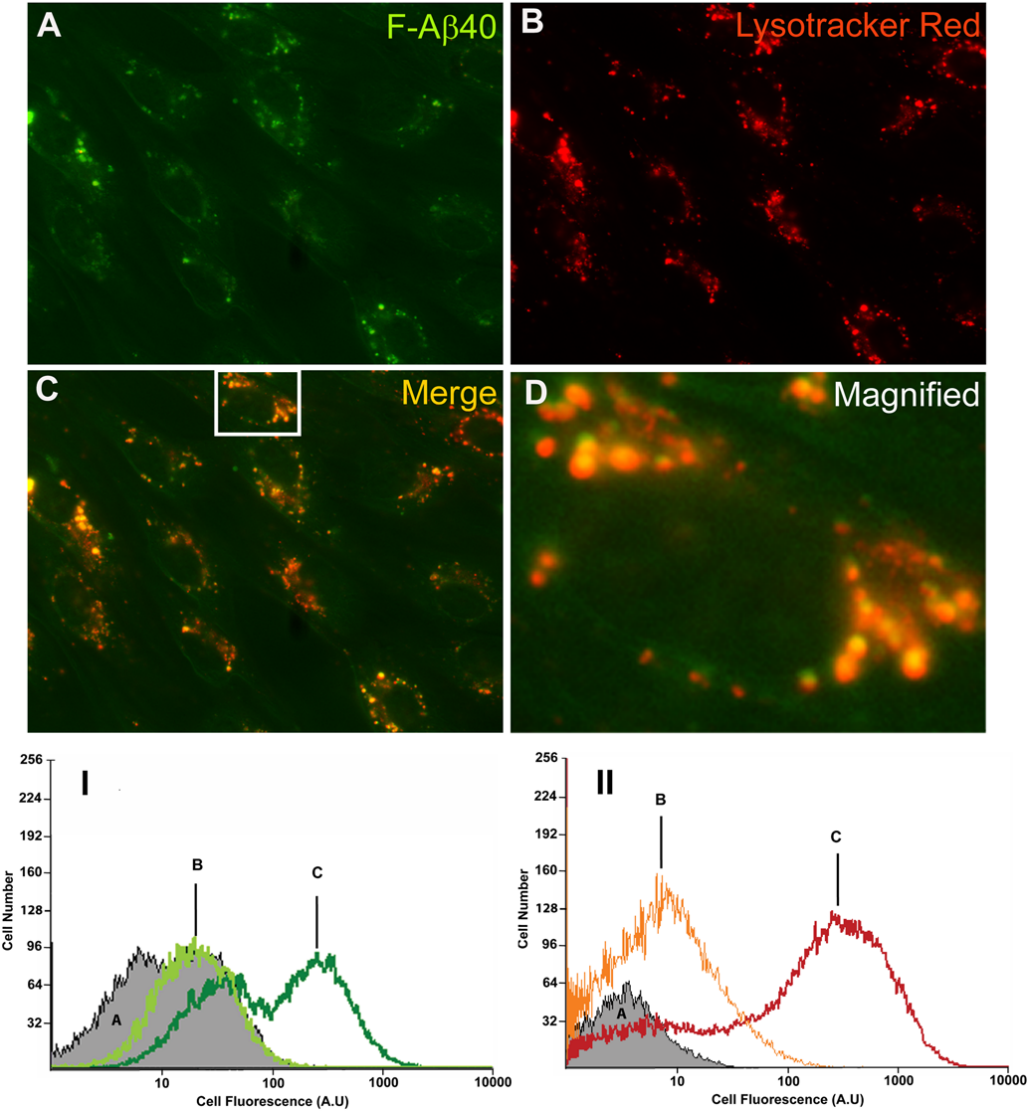
A–D: F-Aβ40 uptake into the acidic compartments of bovine brain microvascular endothelial (BBME) cells labeled by Lysotracker Red® (60×). (A) Uptake of F-Aβ40 (B) Uptake of Lysotracker Red®; (C) Superimposition of images A and B; (D) A magnified portion of image C (enclosed in the white rectangle) to show co-localization of both fluorophores. I–II: Histograms of fluorescence intensity in BBME cells treated with (I) F-Aβ40: (A) Untreated control, (B) at 4°C, (C) at 37°C; and (II) AF633-Trf: (A) Untreated control, (B) at 4°C, (C) at 37°C.

In contrast to the observations made hitherto in neuronal cells, BBME cells accumulated F-Aβ40 in the acidic cell organelles stained with Lysotracker Red® (**Figure 11A–D**). In addition, a comparison of fluorescence intensities in BBME cells treated simultaneously with F-Aβ40 and AF633-Trf at 4°C or 37°C revealed that the cellular uptake of both fluorophores decreased significantly at 4°C compared to that at 37°C (**Figure 11I–II**). These results demonstrated that the uptake of F-Aβ40 in BBME cells is temperature dependent, which is contrary to the observation made in neuronal cells.

## Discussion

Mounting evidence suggests that amyloid plaques are a down stream reflection of neurotoxicity caused by accumulating Aβ proteins in the cortical and hippocampal neurons. Cellular mechanisms leading to the accumulation of Aβ proteins in the neurons have not been clearly elucidated. Without this knowledge, understanding of how Aβ proteins mediate neurodegeneration remains incomplete. The current study is aimed at bridging this knowledge-gap by a methodical investigation of Aβ40 uptake in mouse brain slices and rat primary hippocampal neurons as well as the internalization of Aβ40 and Aβ42 in neuron like PC12 cells.

Additionally, the involvement of cerebral vasculature in AD pathogenesis is widely acknowledged. The BBB is believed to play a vital role in regulating Aβ40 and Aβ42 concentrations in the brain interstitial fluid. The BBB may modulate Aβ40/42 ratio that influence the formation of vascular amyloid versus parenchymal amyloid paques. Earlier reports have established that the Aβ proteins are internalized by the BBB endothelial cells via receptor mediated endocytosis [16], [19], [20]. The current study compares and contrasts the mechanisms involved in the neuronal and cerebrovascular endothelial cell uptake of the amyloid proteins.

Although, Aβ42 is more pathogenic than Aβ40, F-Aβ42 cellular internalization was not investigated in detail because: a) F-Aβ42 mostly exists as oligomers that have widely different biophysical properties; as a consequence, it may exhibit heterogeneous cellular interactions. Currently, methods are being developed in our labs to purify each predominant oligomeric species and study its interactions with neurons and endothelial cells; b) in the context of neurovascular etiology of AD, Aβ40 is relevant, because it is a major component of parenchyma plaques and predominates Aβ42 in cerebrovascular amyloid deposits. Therefore, following Aβ40 interactions with the vascular and parenchymal brain compartments could provide vital clues in deciphering the pathogenesis of AD and CAA

In WT mouse brain slices, F-Aβ40 is preferentially taken up by a subpopulation of cortical and hippocampal neurons, which also internalized clathrin mediated endocytotic marker, AF633-Trf. However, F-Aβ40 showed only partial co-localization with AF633-Trf suggesting non-endocytotic uptake of F-Aβ40 along with the internalization via endocytosis reported by other investigators [30], [31]. The colocalization data alone is usually not sufficient to rule out, or for that matter, confirm the involvement of endocytosis in the internalization of F-Aβ40. Therefore, similar uptake studies were conducted on the brain slices at 4°C, which inhibited AF633-Trf uptake without affecting the internalization of F-Aβ40. The results from these studies provide clear evidence that F-Aβ40 is taken up by adult neurons in WT mouse brain slices via non-endocytotic and energy independent process (**Figure 1**). However, these experiments were performed at 15 µg/ml F-Aβ40 concentrations, which may be considered higher than the reported endogenous brain levels of Aβ40. Therefore, more evidence was sought to substantiate this observation.

Aβ40 concentration in the brain interstitial fluid of a 4 month old Tg 2576 transgenic mice (express human amyloid precursor protein) was reported to be 3 ng/ml [32]; whereas, the amount of Aβ40 per gram of brain tissue was reported to be around 3 µg/g [32] and 70 µg/g [33] in 4 and15 month old Tg 2576 transgenic mice, respectively. In AD patients Aβ40 concentrations in CSF were reported to be 2.65±1.25 µg/ml [34], whereas the brain tissue concentrations ranged between 1.07±0.16 µg/g and 66.5±18.7 µg/g (reviewed by Gregory and Halliday, 2005 [35]). This prompts the question: which one of these Aβ40 concentrations is relevant for use as donor concentration in the neuronal uptake studies conducted in vitro? Considering Aβ40 concentration in the brain interstitial fluid without factoring in Aβ40 affinity to the cell membranes, which according to Fick's first law of diffusion will significantly enhance the flux of a permeant across a barrier, could provide an underestimate of Aβ40 concentrations available for neuronal uptake. On the other hand, assuming Aβ40 concentration in the brain tissue as the donor concentration available for neuronal uptake may be an over estimate.

This quandary could be resolved by determining if the neuronal uptake of Aβ40 at lower donor concentrations, representative of the concentrations in brain interstitial fluid, is linearly related to the uptake at higher Aβ40 concentrations prevailing in the brain tissue. In vitro brain slice uptake studies clearly demonstrated a linear relationship between ^125^I-Aβ40 uptake at very low donor concentrations (5 ng/ml) and the uptake at higher donor concentrations of 450 or even 900 ng/ml (**Figure 2I**). Moreover, the magnitude of uptake throughout the concentration range was found to be similar at 37 and 4°C. Also, the uptake is not significantly affected by an endocytotic inhibitor, dansyl cadaverine (**Figure 2II**). Although, brain slice is a complex tissue comprising of a variety of cells, the uptake of ^125^I-Aβ40 in the brain slices could be correlated to the neuronal uptake, because the confocal images presented in **Figure 1** clearly demonstrate that F-Aβ40 is primarily internalized by the cortical and hippocampal neurons in the brain slice. Furthermore, washing the brain slices with acidified KRB, successfully removed most of the background fluorescence without significantly affecting the intra-neuronal fluorescence.

In addition to the observations made in brain slices, the linearity of F-Aβ40 uptake by differentiated PC12 cells has been established through flow cytometry (**Figure 2III**).

Taken together, these results strongly suggest that:

a) Aβ40 could be internalized by neurons via passive diffusion. This inference is groundbreaking as it contests the current belief that Aβ40 is internalized via endocytosis (reviewed by LaFerla et al., 2007); b) Aβ40 uptake by neurons or by neuron like PC-12 cells is linear over a wide concentration range (5 ng/ml–15 µg/ml). This observation provides rationale for using higher concentrations of F-Aβ40 (15 µg/ml) to investigate the mechanism of F-Aβ40 uptake.

The conventional mode of cellular entry for proteins like Aβ40 is endocytosis, which involves adsorption of the protein to plasma membrane or membrane-bound receptor, followed by energy-dependent uptake through the formation of a vesicle. Thereafter, the protein is processed in the acidic compartments for destruction or recycling. Endocytosis could be carried out by three known energy-dependent mechanisms such as: clathrin-mediated endocytosis; caveolae-mediated endocytosis; and the endocytosis independent of clathrin and caveolin. A battery of confocal microscopy and flow cytometry studies were employed to examine the involvement of these processes in the uptake of F-Aβ40 by neuronal cells.

Localization of a significantly large portion of F-Aβ40 in the cytoplasm of PC12 cells and RPH neurons, distinctly separate from the acidic cell organelles labeled by lysotracker, is indicative of non-endocytotic uptake (**Figure 3**). Confocal imaging of differentiated PC12 cells incubated with F-Aβ40 and AF633-Trf (a marker of clathrin-mediated endocytosis that localizes primarily in the early endosmes) did not show significant co-localization of F-Aβ40 with the marker at 15, 30, 45, or 60 min after incubation (**Figures 4** **&** **5**). Moreover, the z-stack projections of the PC12 cells treated with F-Aβ40 and AF633-Trf not only confirmed the cytosolic distribution of F-Aβ40 but also showed the accumulation of F-Aβ40 and AF633-Trf at different cellular locations (**Figure 5D**). The uptake experiments conducted with Dil labeled low density lipoprotein (Dil-LDL), a clathrin-mediated endocytosis marker that labels secondary endosomes, demonstrated that only a portion of internalized F-Aβ40 accumulates in the secondary endosomes (**Figure 6A–D**). Similar experiments conducted in the presence of AF647-CT provided evidence against the contribution of caveolae-mediated endocytosis in the internalization of F-Aβ40 by PC12 cells (**Figure 7A–D**). In addition, the conditions that can inhibit clathrin-mediated endocytosis (hypotonic shock followed by treatment with K^+^ depleted salt solution) and caveolae-mediated endocytosis (treatment with filipin) inhibited the uptake of AF633-Trf (**Figure 4E–G**) and AF647-CT (**Figure 7E–G**), respectively, but not F-Aβ40. These results provide strong evidence for the non-endocytotic uptake of F-Aβ40.

Experiments conducted to evaluate the cellular uptake of F-Aβ40 (**Figure 8**) and F-Aβ42 (**Figure 9**) at 4°C or under ATP depleted conditions demonstrated their energy independent internalization by differentiated PC12 cells. These observations were based on: direct visualization of fixed or live cells using confocal microscopy; and by quantifying the cellular fluorescence in live cells using flow cytometry. AF633-Trf was used as negative control in both analyses. Flow cytometry allows for a quantitative measurement of internalized protein without running into artifacts caused by cell fixation. However, this analytical technique cannot differentiate membrane bound protein from the internalized protein. Therefore, the cells were trypsinized before the flow cytometry analysis to remove any protein bound to cell membranes. It was previously reported that trypsin treatment effectively removes cell surface-bound Aβ proteins [36] and transferrin [37]. The information obtained from flow cytometry can be correlated with confocal micrographs, which clearly showed the perinuclear localization of F-Aβ40, F-Aβ42, and AF633-Trf. Interestingly, the flow cytometry data as well as the confocal micrographs showed significantly greater localization of F-Aβ40 in ATP depleted cells compared to the normal cells at 37°C. Although the uptake of F-Aβ40 via energy-independent pathway is expected to be similar in normal and ATP depleted cells, inhibition of proteolytic enzymes such as insulin degrading enzyme and nepralysin that are known to degrade Aβ40 might be responsible for the greater F-Aβ40 accumulation in ATP depleted cells.

Non-endocytotic and energy independent F-Aβ40 uptake discovered in the adult hippocampal neurons of WT mouse brain slices as well as in differentiated PC12 cells was also verified in RPH neurons (**Figure 10**). F-Aβ40 accumulated in these neurons without co-localizing with endocytotic marker AF633-Trf. Partial co-localization of Aβ42 with clathrin and caveolae endocytotic markers has also been reported previously [38]. Moreover, conditions that prevent endocytotic uptake of AF633-Trf, 4°C and ATP depletion, did not affect F-Aβ40 uptake.

However, BBB endothelial cells (BBME cells), a major constituent of the neurovascular unit believed to play a critical role in neurodegenerative diseases like AD and vascular dementia, internalized F-Aβ40 via endocytotic and energy dependent pathways (**Figure 11**). This inference was drawn from two crucial observations: a) in BBME cells, almost the entire amount of internalized F-Aβ40 accumulated in the acidic cell compartments, which suggests endocytotic uptake; and b) like the uptake of endocytotic marker AF633-Trf, the uptake of F-Aβ40 was inhibited at 4°C and under ATP depleted conditions.

Energy independent uptake of cell penetrating peptides in various cell types has been proposed previously [39]. But the energy independent uptake of proteins specific to a particular cell type is very unusual. Nevertheless, the possibility of Aβ40 displaying such attribute may not come as a surprise if the recent literature describing the biophysical and physiological behavior of this protein is carefully examined.

In attempting to study the neuronal internalization of Aβ40 and 42, researchers in the past have encountered non-saturable and energy independent uptake of these proteins [17]. This atypical behavior has also been reported with other β-sheet forming proteins such as human calcitonin [40]. It was argued that the β-sheet structure could facilitate the interaction of the protein with the plasma membrane and enhance its passive diffusion across the cellular barrier [40]. Several researchers have reported the ability of Aβ40 to intercalate in the hydrocarbon core of the neuronal membrane and increase its fluidity [21], [41]–[44]. After attaining higher concentrations in the neuronal membrane, Aβ40 could passively diffuse to a region of lower concentration, most likely the neuroplasm. These biophysical interactions of Aβ40 with the plasma membrane were reported to be influenced by the membrane lipid composition [45], which could change significantly with cell type. In addition to the expression of Aβ40 receptors, differences in the plasma membrane lipid composition of neurons and BBB endothelial cells might be responsible for their differential internalization of Aβ40. Also, the membrane lipid composition may change drastically between normal and AD subjects [46], which might explain the vulnerability of cortical and hippocampal neurons to Aβ toxicity in AD.

In conclusion, the current study demonstrates that Aβ40 is internalized by neurons primarily via non-endocytotic and energy independent pathways, most likely due to its ability to biophysically interact with the neuronal membrane. A significant proportion of internalized Aβ40 is located outside of the endosomal or lysosomal compartments; as a consequence, the protein could accumulate in the neuroplasm without degradation and subsequently aggregate to form fibrils. In contrast, BBME cells exhibit energy dependent uptake of Aβ40 and accumulate the protein in acidic cell organelle such as endosomes and lysosomes, which is indicative of endocytotic uptake. Such a phenomenal difference in the internalization of Aβ40 between neurons and BBB endothelial cells may provide essential clues to understanding how various cells can differentially regulate Aβ proteins and help explain the vulnerability of cortical and hippocampal neurons to Aβ toxicity.

## Materials and Methods

### Synthesis of fluorescein labeled human Aβ40 (F-Aβ40)

F-Aβ40 was synthesized on an ABI 433 peptide synthesizer (Foster City, CA) with Val-NovaSyn TGA resin (Calbiochem-Novabiochem, San Diego, CA) employing HBTU activation and synthesis protocols recommended by the manufacturer. After the final deprotection of the N-terminal Fmoc group, a two equivalent excess of NHS-fluorescein (Pierce, Rockford, IL) was dissolved in 6 ml of dimethylformamide (DMF) and added to the resin saturated with 12% diisopropylethylamine/dichloromethane (DIEA/DCM). The resin slurry was mixed overnight at room temperature, followed by several washes with DMF and DCM. The efficiency of fluorescein addition was confirmed by a negative ninhydrin reaction. The protein was purified by reverse-phase HPLC using a heated 250×21.2 mm C18 Jupiter column (Phenomonex Corporation, Torrance, CA). The molecular weight of the protein was confirmed by electrospray ionization mass spectrometry (Sciex API 165).

### Radioiodination of Aβ40

Human Aβ40 (500 µg) was labeled with ^125^I using the chloramine-T procedure as described previously [47]. Free radioactive iodine was separated from the radiolabeled Aβ40 by dialysis against 0.01 M phosphate buffered saline at pH 7.4 (Sigma-Aldrich Co., St. Louis, MO). Purity of ^125^I-Aβ40 was determined by trichloroacetic acid precipitation method.

### Animals

Wild type (WT) mice (B6/SJL) were obtained from The Jackson Laboratory (Bar Harbor, ME) at 6–8 weeks of age. The mice were housed in a virus-free barrier facility under a 12-hr light/dark cycle, with ad libitum access to food and water. All the experimental procedures were performed in accordance with the NIH Guide for the Care and Use of Laboratory Animals using protocols approved by the Mayo Institutional Animal Care and Use Committee.

### Cell cultures

Rat PC12 cells were plated on glass coverslips or coverslip-bottomed dishes and cultured in DMEM supplemented with: 10% fetal bovine serum, 5% horse serum, 4 mM glutamine, 200 units/ml penicillin, 200 µg/ml streptomycin, and 100 ng/ml nerve growth factor (NGF) (Harlan, Indianapolis, IN) at 37°C under 5% CO_2_. Uptake studies were conducted 5–7 days after plating, when the cells were well differentiated and the neurite growth was prominent.

Rat primary hippocampal (RPH) neurons were isolated from the hippocampii of 18-day-old embryonic Sprague Dawley rat brains (Neuromics, Edina, MN). The hippocampii were dispersed using a fire polished Pasteur pipette and plated on poly-D-lysine (Sigma-Aldrich, St. Louis, MO) coated glass cover slips in B-27 neurobasal medium containing 0.5 mM glutamine and 25 µM glutamate (Invitrogen, Carlsbad, CA). The neuronal cells were grown under 5% CO_2_ in an incubator maintained at 37°C until differentiation.

Bovine brain microvascular endothelial (BBME) cells were obtained frozen from Cell Applications Inc. (San Diego, CA) and were grown in 75 cm^2^ cell culture flasks coated with collagen. The growth medium was made of equal parts DMEM and F-12 Ham nutrient mix containing amphotericin B (2.5 µg/ml), HEPES (10 mM), donor horse serum (10%), penicillin (100 units/ml), and gentamicin sulphate (15 µm/ml). After reaching 70–80% confluency, the cells were harvested, seeded (80,000 cells/cm^2^) on six-well cell culture plates coated with 0.01% rat tail collagen, and grown under 5% CO_2_ at 37°C.

### Brain slices experiments

After the mice were sacrificed with an overdose of sodium pentobarbital (200 mg/kg, IP), the brains were removed from the cranial cavity and sliced with tissue chopper (Stoelting, Wood Dale, IL) into 1 mm thick slices containing cortex and hippocampus.

#### Localization of F-Aβ40 and Alexa Fluor 633 labeled transferrin (AF633-Trf)

Following the equilibration in KRB for 30 min at 37°C or 4°C, the brain slices were incubated in KRB containing 15 µg/ml F-Aβ40 and 20 µg/ml AF633 labeled transferrin (AF633-Trf) (Invitrogen-Molecular Probes, Carlsbad, CA), a clathrin mediated endocytosis marker, for 30 minutes at 37°C or 4°C. The incubated brain slices were washed in acidified KRB (pH = 5.0±0.2), rinsed 3 times with ice-cold KRB, and imaged.

#### Uptake of ^125^I-Aβ40 in brain slices

After pre-incubating in KRB for 30 min at 4°C or at 37°C, with or without 1 mM dansyl cadaverine (DC), each brain slice was incubated in 1 ml KRB containing a different concentration of ^125^I-Aβ40 ranging between 5–900 ng/ml for 15 min at 4°C or 37°C. The brain slices pre-incubated with 1 mM DC was incubated with 450 ng/ml at 37°C. At the end of the experiment, all the brain slices were washed in acidified KRB (pH = 5.0±0.2), rinsed 3 times with ice-cold KRB, and assayed for the radioactivity in a dual channel gamma counter (Perkin-Elmer, Waltham, MA).

### Effect of donor concentration on the uptake of F-Aβ40 by PC12 cells

The PC12 cells were dispersed in growth medium containing 100 ng/ml NGF (Harlan Bioproducts, Indianapolis, IN) and seeded at a density of 50,000/well in 6-well plates. On the seventh day, following sets of experiments were conducted on the differentiated PC-12 cells:

- To determine the linearity of F-Aβ40 uptake, PC-12 cells were pre-incubated in serum free DMEM for 30 min at 37°C and then incubated with DMEM solution containing 3–15 µg/ml F-Aβ40 for 30 min at 37°C.
- To determine the saturability of F-Aβ40 uptake, the PC-12 cells were pre-incubated in 150 µg/ml of unlabeled Aβ40 for 30 min at 37°C, followed by a 30 min incubation in the same solution spiked with 15 µg/ml F-Aβ40.

At the end of these experiments, the cells were dissociated with trypsin and centrifuged at 2000 ×g. The cell pellet was washed with and re-suspended in ice cold PBS and analyzed by flow cytometry.

### Cellular localization of F-Aβ40 or F-Aβ42

At the conclusion of the following experiments, cellular localization of F-Aβ40 and other markers was examined by wide field or laser confocal microscopy:

#### Localization of F-Aβ40 in acidic organelles

After pre-incubating in DMEM for 15 min at 37°C, the PC12 cells, RPH neurons, or BBME cells grown on glass coverslips or coverslip-bottomed dishes (MatTek, Ashland, MA) were incubated in DMEM containing 15 µg/ml F-Aβ40 and 75 nM Lysotracker Red® (Invitrogen-Molecular Probes, Carlsbad, CA) for 30 min at 37°C. Thereafter, the cells were washed 3 times with ice-cold PBS and imaged.

#### Characterizing endocytotic mechanisms

Role of endocytosis in the uptake of F-Aβ40 or F-Aβ42 by differentiated PC12 cells and/or RPH neurons was evaluated through the following experiments.

#### Clathrin-mediated endocytosis

Following the pre-incubation with DMEM for 30 min at 37°C, PC12 cells or RPH neurons were incubated in DMEM containing 15 µg/ml F-Aβ40 or F-Aβ42 and 20 µg/ml AF633-Trf or 15 µg/ml of Dil labeled low density lipoprotein (Dil-LDL) for 30 min at 37°C. The cells were washed three times with ice-cold PBS and imaged live or fixed in 3.7% paraformaldehyde.

Alternatively, PC12 cells were pre-incubated for 30 min under potassium free hypotonic conditions, which were reported to inhibit clathrin mediated endocytosis [28]. Potassium depletion was carried out as described previously [28]. Briefly, differentiated PC12 cells were shocked in a hypotonic medium (salt solution/water, 1∶1) at 37°C for 5 min. Then the hypotonic medium was replaced with an isotonic K^+^-free salt solution in which the cells were pre-incubated for 30 min. The cells were then incubated for 30 min at 37°C in DMEM containing 15 µg/ml F-Aβ40 and 20 µg/ml AF633-Trf, washed three times with ice-cold PBS, and imaged.

#### Caveolae-mediated endocytosis

PC12 cells plated on glass coverslips were pre-incubated with DMEM for 30 min at 37°C. Subsequently, they were incubated in DMEM containing 15 µg/ml F-Aβ40 and 5 µg/ml Alexa Flour 647 labeled Cholera toxin (AF647-CT) (Invitrogen-Molecular Probes, Carlsbad, CA), a specific marker of caveolae internalization, for 30 min at 37°C. At the end of the experiment, the cells were washed three times with ice-cold PBS, fixed in 3.7% paraformaldehyde, and imaged.

Alternatively, PC12 cells were pre-incubated for 30 min in DMEM containing filipin (5 µg/ml) (Cayman Chemical Company, Ann Arbor, MI), a sterol-binding agent that selectively inhibits caveolae invagination without affecting the function of clathrin-coated pits [29], followed by incubation in DMEM containing 15 µg/ml F-Aβ40 and 5 µg/ml AF647-CT for 30 min at 37°C. At the end of these experiments, the cells were fixed in 3.7% paraformaldehyde and imaged.

### Effect of temperature and energy on F-Aβ40 uptake

To examine the effect of temperature and cellular energy on the internalization of F-Aβ40 and F-Aβ42, the uptake studies were conducted at 4°C or under ATP depleted conditions. In the studies conducted at 4°C, steps outlined previously to investigate clathrin mediated endocytosis were repeated, but at 4°C. In ATP depletion studies, the PC12 cells or RPH neurons were pre-incubated for 30 min in glucose free DMEM containing 0.1% sodium azide and 50 mM 2-deoxy-D-glucose followed by the incubation with glucose free DMEM containing 20 µg/ml AF633-Trf and 15 µg/ml F-Aβ40 for 30 min at 37°C.

In addition, flow cytometry studies were conducted to quantify the uptake of F-Aβ40 or F-Aβ42 and AF633-Trf by PC12 cells and BBME cells at 4°C or under ATP depleted conditions. At the end of these flow cytometry experiments, the cells were washed three times with ice-cold PBS, trypsinized, and centrifuged at 2000 ×g. The cell pellet was washed with and re-suspended in ice cold PBS, and analyzed.

### Microscopy

#### Wide field Microscopy

The localization of various fluorophores in live cells was investigated with a Nikon Eclipse 80-i fluorescent microscope equipped with FITC (λex, 465–495 nm; λem, 515–555 nm; dichroic splitter, 505 nm) and rhodamine (λex, 530–560 nm; λem, 600–660 nm; dichroic splitter, 595 nm) filters (Nikon Instruments Inc., Melville, NY). Images were captured with a Hamamatsu ORCA-ER CCD camera using a constant exposure time at each filter combination.

#### Confocal Microscopy

Imaging of the brain slices mounted on glass coverslips or the live cells grown on coverslip-bottom culture dishes was conducted using Axiovert 100 M microscope equipped with LSM 510 system (Carl Zeiss MicroImaging, Inc., Thornwood, NY). F-Aβ40 or F-Aβ42 was excited by the 488 nm line of a 200 mW argon ion laser and the emitted fluorescence was detected at wavelengths above 505 nm. Alexa Fluor 633 was excited by the 633 nm line of a 15 mW helium–neon ion laser and the emitted fluorescence signal was collected at wavelengths above 650 nm. Lysotracker Red® was visualized with 543 nm line of HeNe laser and a BP filter 560–615. Imaging of the cells fixed with 3.7% paraformaldehyde and mounted in Gelvatol was conducted on Olympus Fluoview 1000 laser scanning confocal system (Olympus America Inc., Center Valley, PA) based on Olympus IX81 inverted microscope equipped with Olympus UPlanApo 40× 1.00 NA oil objective. F-Aβ40 was imaged using the 488-nm line of a 150 mW multi-line Melles-Griot argon-ion laser with emission collection ranging from 520–539 nm. AF647-CT or AF633-Trf was imaged using a 5 mW 633 nm Melles-Griot argon-ion laser with emission collection ranging from 650–750 nm. Photomultiplier, gain, offset, and confocal aperture settings were maintained the same for all images.

### Flow cytometry

FACScan (Becton Dickinson FACS canto, San Jose, CA) was equipped with 488 nm laser and 530/30 band-pass filter to analyze F-Aβ40 or F-Aβ42 as well as 633 nm laser and 660/20 band-pass filter to analyze AF633-Trf. Each unfixed cell sample was observed to have two distinct populations of cells, one with low and the other with high forward scatter. When treated with live (calcein AM) and dead (ethidium homodimer) cell markers (Invitrogen-Molecular Probes, Carlsbad, CA), the population of cells with high forward scatter contained more than 90% viable cells. The cellular fluorescence from this cell population was presented as histograms along with relevant statistical values such as geometric mean and coefficient of variance.

## Acknowledgments

We thank: Dr. Dan McCormick and Jane A. Petersen for extending their technical expertise in synthesizing the Aβ40; and fluorescein labeled Aβ40 and 42; Geoffry L. Curran for his assistance in harvesting brain tissue from mice and ^125^I labeling of Aβ40; Jarred Nesbitt and Dr. Anthony Windebank for providing PC12 cells; and Jennifer Scott for her excellent secretarial assistance.
